# Lung-Specific Extracellular Superoxide Dismutase Improves Cognition of Adult Mice Exposed to Neonatal Hyperoxia

**DOI:** 10.3389/fmed.2018.00334

**Published:** 2018-12-10

**Authors:** Bradley W. Buczynski, Nguyen Mai, Min Yee, Joshua L. Allen, Landa Prifti, Deborah A. Cory-Slechta, Marc W. Halterman, Michael A. O'Reilly

**Affiliations:** ^1^Department of Environmental Medicine, University of Rochester, Rochester, NY, United States; ^2^Department of Neurology, University of Rochester, Rochester, NY, United States; ^3^Department of Pediatrics, School of Medicine and Dentistry, University of Rochester, Rochester, NY, United States

**Keywords:** anti-oxidants, hyperoxia, neonatal, neurocognitive, long-term consequences, mice, pulmonary

## Abstract

Lung and brain development is often altered in infants born preterm and exposed to excess oxygen, and this can lead to impaired lung function and neurocognitive abilities later in life. Oxygen-derived reactive oxygen species and the ensuing inflammatory response are believed to be an underlying cause of disease because over-expression of some anti-oxidant enzymes is protective in animal models. For example, neurodevelopment is preserved in mice that ubiquitously express human extracellular superoxide dismutase (EC-SOD) under control of an actin promoter. Similarly, oxygen-dependent changes in lung development are attenuated in transgenic *Sftpc*^*EC*−*SOD*^ mice that over-express EC-SOD in pulmonary alveolar epithelial type II cells. But whether anti-oxidants targeted to the lung provide protection to other organs, such as the brain is not known. Here, we use transgenic *Sftpc*^*EC*−*SOD*^ mice to investigate whether lung-specific expression of EC-SOD also preserves neurodevelopment following exposure to neonatal hyperoxia. Wild type and *Sftpc*^*EC*−*SOD*^ transgenic mice were exposed to room air or 100% oxygen between postnatal days 0–4. At 8 weeks of age, we investigated neurocognitive function as defined by novel object recognition, pathologic changes in hippocampal neurons, and microglial cell activation. Neonatal hyperoxia impaired novel object recognition memory in adult female but not male mice. Behavioral deficits were associated with microglial activation, CA1 neuron nuclear contraction, and fiber sprouting within the hilus of the dentate gyrus (DG). Over-expression of EC-SOD in the lung preserved novel object recognition and reduced the observed changes in neuronal nuclear size and myelin basic protein fiber density. It had no effect on the extent of microglial activation in the hippocampus. These findings demonstrate pulmonary expression of EC-SOD preserves short-term memory in adult female mice exposed to neonatal hyperoxia, thus suggesting anti-oxidants designed to alleviate oxygen-induced lung disease such as in preterm infants may also be neuroprotective.

## Introduction

Supplemental oxygen (hyperoxia) used to treat preterm infants in respiratory distress has beneficial and harmful effects. Although used to sustain life, it can cause oxidative damage and inflammation when the production of cytotoxic reactive oxygen species (ROS) exceeds the antioxidant capacity of the developing lung. Preterm infants are particularly sensitive to oxidative stress because maturation of anti-oxidant defenses in the lung does not occur until later in gestation and they have an impaired ability to stimulate expression of anti-oxidant molecules at birth (1). They are also systemically sensitive to oxidative stress. Preterm infant plasma has higher levels of glutathione disulfide compared to adult plasma and can oxidize pulmonary surfactant phospholipids (2). Preterm infant cord blood contains high levels of non-protein bound free iron, which can act as a pro-oxidant by producing highly toxic hydroxyl radical via Fenton chemistry (3). It is generally accepted that this enhanced sensitivity to oxygen and oxidative stress is responsible for why preterm infants are at increased risk for reduced lung function, being re-hospitalized following a respiratory viral infection, and developing airway wheezing later in life. Hence, supplemental oxygen contributes significantly to respiratory morbidity in an oxygen-sensitive preterm infant.

The preterm brain is also sensitive to ROS, perhaps even more than the lung, because it has a high rate of oxygen consumption, lacks the anti-oxidants superoxide dismutase and glutathione peroxidase, and contains high levels of oxidizable polyunsaturated fatty acid (4). The period of greatest vulnerability during brain development coincides with the “brain growth spurt,” which begins around the 6 month of gestation in humans and continues for several years after birth (5). Further, preterm infants are at risk for developing periventricular leukomalacia, characterized by periventricular necrosis, cystic formation, and diffuse cerebral white matter injury. This may explain why survivors of preterm birth also face increased risk of neurodevelopmental delay that can affect learning, behavior, motor skills, memory, and the incidence of cerebral palsy (6, 7). Indeed, poor neurodevelopmental outcomes are observed in preterm children at 2 and 3 years of age who had the lowest and highest cumulative amount of regional cerebral oxygen saturations on day 1 of life (8). Fluctuating levels of cerebral oxygen created when the infant de-saturates and is re-oxygenated may therefore be responsible for these deficits in brain development and cognitive function later in life.

It is challenging to model preterm birth in small animals like mice or rats, however, these rodents have helped our understanding of how the transition to excess oxygen at birth alters postnatal development of the lung, brain, and other organs. Analogous to people born preterm, young adult rodents exposed to hyperoxia at birth exhibit reduced lung function, hyperactive airways, altered host response to viral infections, and cardiovascular disease (9–11). They also exhibit adverse brain development and cognitive deficits. For example, young adult mice exposed to hyperoxia between postnatal days (PND) 1 and PND14 show abnormal spatial and recognition memory associated with atrophy in hippocampal subfields CA1 and CA3 (12). It may do so in rats and mice by inhibiting proliferation and promoting apoptosis of both neurons and oligodendrocyte progenitors (5, 13, 14). Studies have also shown the hyperoxia can also have potent effects on the inflammatory milieu within the CNS. For example, exposure to 80% oxygen from PND 6–8 results in sustained, age-dependent changes in GFAP+ astrocytes within the white matter (14). Similarly, protection against hyperoxia-induced white matter conveyed by minocycline was associated with a reduction in the activation of Iba1+ microglia (15). Hyperoxia also increases iNOS expression in microglial cells of neonatal rat brains. Elevated levels of iNOS could increase oxidative/nitrosative stress in sensitive cells (16). Hence, high oxygen exposure in newborn animals can permanently disrupt brain development by increasing oxidative stress and inflammatory injury to developing white matter (17).

It is therefore not entirely surprising that antioxidants can block or attenuate oxygen-dependent changes in lung and brain development. Preterm infants administered recombinant human copper-zinc (CuZn)-SOD intratracheally show improved pulmonary function at 1 year of corrected age (18, 19). CuZn-SOD also been shown to improve lung gas exchange and prevented the development of pulmonary hypertension in preterm ventilated lambs (20, 21). Similarly, overexpression of extracellular (EC)-SOD in alveolar epithelial type 2 cells (AEC2) of transgenic *Sftpc*^*EC*−*SOD*^ mice inhibited oxygen-dependent changes in alveolar epithelial cell proliferation and lung development (22). EC-SOD also preserved ability of the oxygen-exposed lung to effectively regenerate the respiratory epithelium following influenza A virus infection (23). Likewise, neonatal hyperoxia-induced neuronal apoptosis and brain injury is significantly diminished in transgenic mice that ubiquitously express an extra copy of EC-SOD under control of the actin promoter (24). Because EC-SOD catalyzes dismutation of the superoxide radical to hydrogen peroxide and water, these studies using transgenic mice over-expressing EC-SOD suggest that neonatal hyperoxia disrupts lung and brain development via the production of superoxide.

Despite the appreciation that superoxide is a critical mediator of oxygen toxicity to the lung and the brain, the site of superoxide production is not entirely clear. Superoxide is highly reactive and promotes tissue injury where it is produced. Not surprisingly, anti-oxidants targeted to the lung protect the developing lung against hyperoxia. But, how high oxygen in the lung perturbs brain development via the production of superoxide is less clear. Although FiO_2_ is high in the lungs of preterm infants treated with supplemental oxygen, pAO_2_ levels are aggressively monitored to reduce oxidant injury to other tissues, particularly the developing brain. Moreover, the small increase in arterial oxygen saturations created during hyperoxia does not significantly increase oxygen delivery to the brain because oxygen reduces cerebral blood flow (25). Hence, the source of superoxide produced during hyperoxia that affects neurodevelopment has not been well established. We hypothesized that the lung is the primary source of superoxide that damages the brain because it is has the highest amount of oxygen during exposure. Here, we use *Sftpc*^*EC*−*SOD*^ mice to determine whether pulmonary expression of EC-SOD can mitigate oxygen-dependent changes in cognition acquired during the perinatal period. Our studies reveal the unexpected and novel finding that modification of the redox environment in the lung can protect against both behavioral and structural correlates of neonatal hyperoxia-induced brain injury.

## Materials and Methods

### Exposure of Mice to Hyperoxia

C57BL/6J mice (wild type) and *Sftpc*^*EC*−*SOD*^ transgenic (Tg) mice on the same genetic background were used for this study. Newborn mice were exposed to room air or 100% oxygen (hyperoxia) between postnatal days (PND) 0 and 4 (23). Dams were cycled between litters exposed to room air and hyperoxia every 24 h during the exposure to protect against acute oxygen toxicity. On PND 4, hyperoxia-exposed mouse pups were returned to room air, where they remained until behavioral assessment at 8–10 weeks of age. Mice were housed in micro-isolator cages in a specified pathogen-free environment according to a protocol approved by the University Committee on Animal Resources at the University of Rochester (UCAR # 20017-121R), and they were provided food and water *ad libitum*.

### Western Blot Analysis

Frozen lungs and whole brain from newborn (PND 4) and young adult (8–10 weeks of age) mice were homogenized in lysis buffer and protein concentration in supernatants was measured as previously described (26). Protein samples were diluted in Laemmli buffer, separated by SDS-PAGE, and then transferred to polyvinylidene difluoride membranes (Pall Life Sciences, Port Washington, NY, United States). After blocking the membranes with 5% non-fat dry milk, they were incubated overnight with goat anti-mouse SOD3/EC-SOD (1:500 dilution; R & D Systems, Minneapolis, MN, United States), goat anti-human SOD3/EC-SOD (1:500 dilution; R & D Systems), or rabbit anti-mouse β-actin (1:1,000 dilution; Sigma, St. Louis, MO, United States) antibodies. The membranes were then incubated with the appropriate horseradish peroxidase-conjugated secondary antibody (anti-goat at 1:5,000 and anti-rabbit at 1:5,000; Southern Biotechnology) and immune complexes were visualized by enhanced chemiluminescence (ECL kit, GE Life Sciences, Piscataway, NJ, United States).

### Novel Object Recognition Memory and Locomotor Activity Procedures

Prior to the start of novel object recognition and locomotor activity testing, young adult mice (8–10 weeks of age) exposed to room air or hyperoxia at birth were moved into housing located in the behavioral testing suite and allowed to acclimate for ~1 week. Novel object recognition testing assesses short-term memory and is premised on the innate preference of rodents for a novel stimulus, such that greater time with a novel object stimulus requires remembering objects previously encountered (27). As previously described (28, 29), male and female mice were individually placed into an acrylic arena containing two similar, fixed objects (non-novel) and were observed for 10 min (learning trial). After a minimum of 1 h, one of the non-novel objects was replaced with a novel object, and mice were placed back into the chamber and observed for 5 min (test trial). Both learning and test trials were video recorded. A recognition index was determined based on the proportion of total time spent in contact with the novel object, while a time per approach index was determined based on the proportion of total time spent approaching the non-novel object. Following completion of the novel object recognition testing, locomotor activity was measured separately in female and male mice in photobeam chambers equipped with a transparent acrylic arena (Med Associates, St. Albans, VT). Jumps, vertical counts, ambulatory episodes, stereotypic counts, and resting time were quantified in three 60-min sessions occurring once per day on three consecutive days as previously described (28, 29).

### Immunohistochemistry

To analyze pathological changes within the CNS, brains were removed after intracardiac perfusion, fixed in 4% paraformaldehyde, and cut in 25-micrometer sections. Sections were washed with PBS, blocked in 10% goat serum in PBS for 1 h at 20°C, and incubated for 18 h at 4°C in the following antibodies: rabbit polyclonal anti-ionized calcium-binding adapter molecule 1 (Iba1; 1:1,000; Wako; Richmond, VA), mouse monoclonal anti-glial fibrillary acidic protein (GFAP; 1:400; Sigma-Aldrich; St. Louis, MO), and rat monoclonal anti-myelin basic protein (MBP; 1:1,000; EMD Millipore; Billerica, MA, United States). After washing with PBS, sections were subsequently incubated for 1 h at 20°C in the following secondary antibodies: Alexa Fluor 594 goat anti-rabbit IgG (H+L), Alexa Fluor 488 goat anti-mouse IgG (H+L), and Alexa Fluor 594 goat anti-rat IgG (H+L) (all at 1:1,000; Life Technologies; Grand Island, NY, United States) and counterstained with Hoechst 3342 (1:1,000; Sigma-Aldrich).

### Imaging and Quantitative Analyses

Brain sections were imaged using an OptiGrid Structured-Light Imaging System (Qioptiq, Fairport, NY, United States) and quantified using ImageJ (http://rsb.info.nih.gov/ij/). For analysis of inflammation around CA1 of the hippocampus, the mean gray values for Iba1 and GFAP were measured 50 micrometers above and below CA1 in matched bregma sections for each mouse. Sholl analysis of microglial arborization was conducted with ≥10 microglia per animal from the same region imaged at 40 times (40X) magnification (30). Neuronal nuclear area was measured by acquiring Z-stack images of the same CA1 region in matched bregma sections. For each image, the outer edges of ≥100 Hoechst-labeled nuclei were traced using a pen-based graphic tablet (Wacom Inc., Vancouver, WA, United States), and nuclear surface area was calculated using ImageJ (31). For skeleton analysis of neuronal fiber sprouting using MBP, 20x images of the dentate gyrus (DG) hilus were acquired from sections stained with MBP for each animal. Using ImageJ, images were made binary, skeletonized, and quantified using the Analyze Skeleton plugin (32). Voxels were classified as “end-point” if they have <2 neighboring voxels, “junction” if they have >2 neighbors, and “slab” if they have precisely 2 neighbors. Branch length was defined as the sum of all slab voxels in each branch converted to micrometers.

### Statistical Analysis

Locomotor activity was assessed by repeated measures analysis of variance (RMANOVA), with between factors of strain and treatment, and a within factor of session number, followed by Fisher's least significant difference *post-hoc* tests as appropriate depending upon confirmation of main effects or interactions. Novel object recognition was assessed by analysis of variance (ANOVA), followed by Fisher's least significant difference *post-hoc* tests. Statistical analyses were carried out using StatView statistical software (Abacus Concepts, Piscataway, NJ, United States). For immunohistochemistry, comparisons were made using two-way ANOVA and Holm-Sidak *post-hoc* test for Iba1 and GFAP mean gray values. Differences in microglial arborization were calculated using repeated measures ANOVA for the number of intersections at each radius from the soma. Gaussian best-fit curves for the distribution of CA1 nuclear area and DG branch length were generated in Prism (Graphpad, La Jolla, CA, United States), and means were compared using the extra sum-of-squares F test. Values are expressed as means ± SE with *P* < 0.05 being considered significant.

## Results

### EC-SOD Is Over-expressed in Lungs but Not Brains of *SFTPC^*EC*−*SOD*^* Transgenic Mice

**Figure 1:**
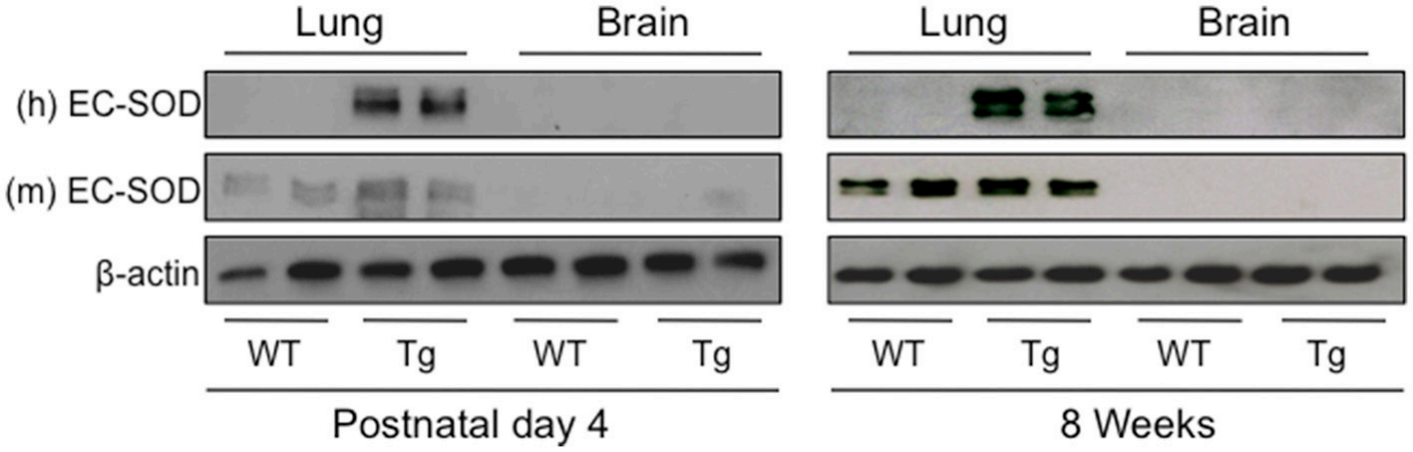
Human EC-SOD protein is detected in the lungs, but not brains, of transgenic *Sftpc*^*EC*−*SOD*^ mice. Lung and brain tissues were harvested on postnatal day 4 and at 8 weeks of age from *wild type* (WT) and *Sftpc*^*EC*−*SOD*^ transgenic (Tg) mice birthed into room air. Lung and brain homogenates were immunoblotted with antibodies to detect human (h) EC-SOD, mouse (m) EC-SOD, and β-actin (loading control). Each lane represents an individual mouse.

Before testing whether over-expression of human EC-SOD in the lung preserves cognition in adult mice exposed to neonatal hyperoxia, we first confirmed human EC-SOD was expressed in the lung but not the brains of transgenic (Tg) mice. Lungs and brains were harvested from PND4 and 8 week-old wild type (WT) and *Sftpc*^*EC*−*SOD*^ transgenic (Tg) mice and immunoblotted with antibodies specific for the Tg human and endogenous mouse EC-SOD protein. In PND4 and 8-week-old mice, human EC-SOD protein was detected in Tg but not WT lung (Figure 1). It was not detected in WT or Tg brain. In contrast, the endogenous mouse EC-SOD protein was detected in both WT and Tg lung but not in brain, which is consistent with the reported low expression of endogenous EC-SOD in the brain (33).

### EC-SOD in the Lung Protects Against Neurodevelopmental Impairment in Mice Exposed to Hyperoxia as Neonates

**Figure 2:**
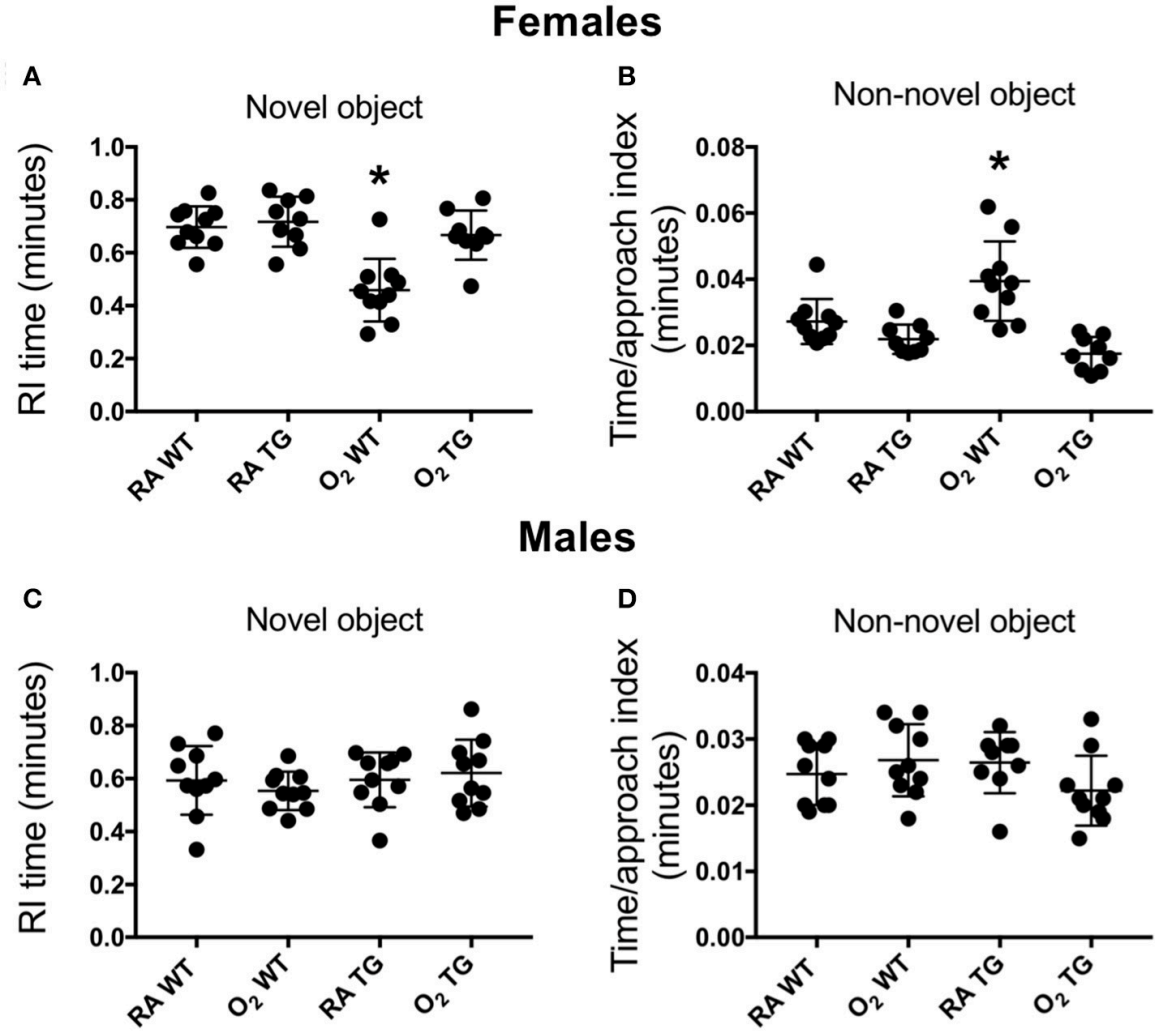
Over-expression of EC-SOD in the lung preserves the ability to learn in adult female mice exposed to neonatal hyperoxia. Novel object recognition testing was evaluated separately in female **(A,B)** and male **(C,D)** adult *wild type* (WT) and *Sftpc*^*EC*−*SOD*^ transgenic (Tg) mice that were exposed to room air (RA) or 100% oxygen (O_2_) between postnatal days (PND) 0–4. A recognition index **(A,C)** based on the proportion of total time spent in contact with the novel object, as well as a time per approach index **(B,D)** based on the proportion of total time spent approaching the non-novel object were measured to assess learning. Neonatal hyperoxia significantly decreased recognition of the familiar object in female WT mice (**A**, ^*^P < 0.0001) but not in Tg mice (**B**, *P* = 0.28). Neonatal hyperoxia significantly increased recognition of the familiar object in female WT (**C**, ^*^P < 0.002) but not in Tg mice (**D**, *P* = 0.25). *n* = 9–10 mice per group. Values from individual mice are represented as circles.

Novel object recognition, a measure of short-term memory was evaluated in adult WT and Tg mice that had been exposed to room air or 100% oxygen between postnatal days 0–4. This time period of oxygen exposure coincides with the saccular phase of lung development in mice, which closely parallels the developmental stage of when preterm human lungs are first exposed to oxygen. Testing was conducted separately in female and male mice. When a recognition index based on time was assessed, exploratory time with the novel object was significantly reduced in female WT mice exposed to hyperoxia as neonates compared to siblings birthed into room air (Figure 2A). In contrast, no differences in the recognition index were observed when female EC-SOD Tg mice exposed to room air or hyperoxia as neonates were compared. Additionally, when a time per approach index was assessed, the amount of time spent approaching the non-novel (familiar) object was significantly increased in female WT mice exposed to hyperoxia as neonates, but not EC-SOD Tg mice, compared to respective siblings birthed into room air (Figure 2B). No differences in novel object recognition were observed in male mice exposed to room air or hyperoxia as neonates (Figures 2C,D).

**Figure 3:**
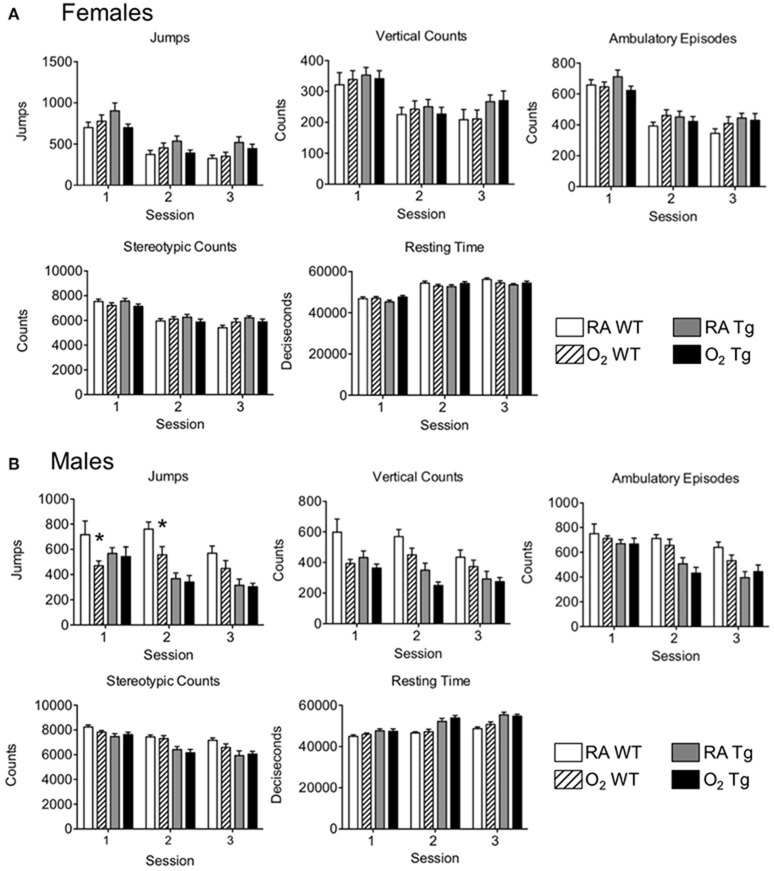
Exposure to neonatal hyperoxia does not affect locomotor activity in adult mice. Locomotor activity testing was evaluated in adult **(A)** female and **(B)** male *wild type* (WT) and *Sftpc*^*EC*−*SOD*^ transgenic (Tg) mice (8–10 weeks of age) exposed to room air (RA) or 100% oxygen (O_2_) during postnatal days (PND) 0–4. Jumps, vertical counts, ambulatory episodes, stereotypic counts, and resting time were quantified in three 60-min sessions occurring once per day on three consecutive days (*n* = 9–10 mice per group, ^*^P < 0.05 when compared to WT mice exposed to room air as neonates).

Following completion of novel object recognition testing, five different parameters of locomotor activity were assessed. Testing was conducted separately in female and male mice. Locomotor function was comparable amongst all female test groups during the first test session and equivalently declined during sessions 2 and 3 (Figure 3A). No oxygen-related differences in locomotor function were observed for any of the parameters assessed between female WT and EC-SOD Tg mice. With the exception of fewer jumps observed in male WT mice exposed to hyperoxia as neonates compared to siblings birthed into room air during sessions 1 and 2 (Figure 3B), trends in locomotor activity among male mice were comparable to those observed for the female mice. Data for individual mice may be found in the Supplementary Table 1.

### EC-SOD in the Lung Blunts Hyperoxia-Induced Neuronal Cell Pathology but Not Neuroinflammation

**Figure 4:**
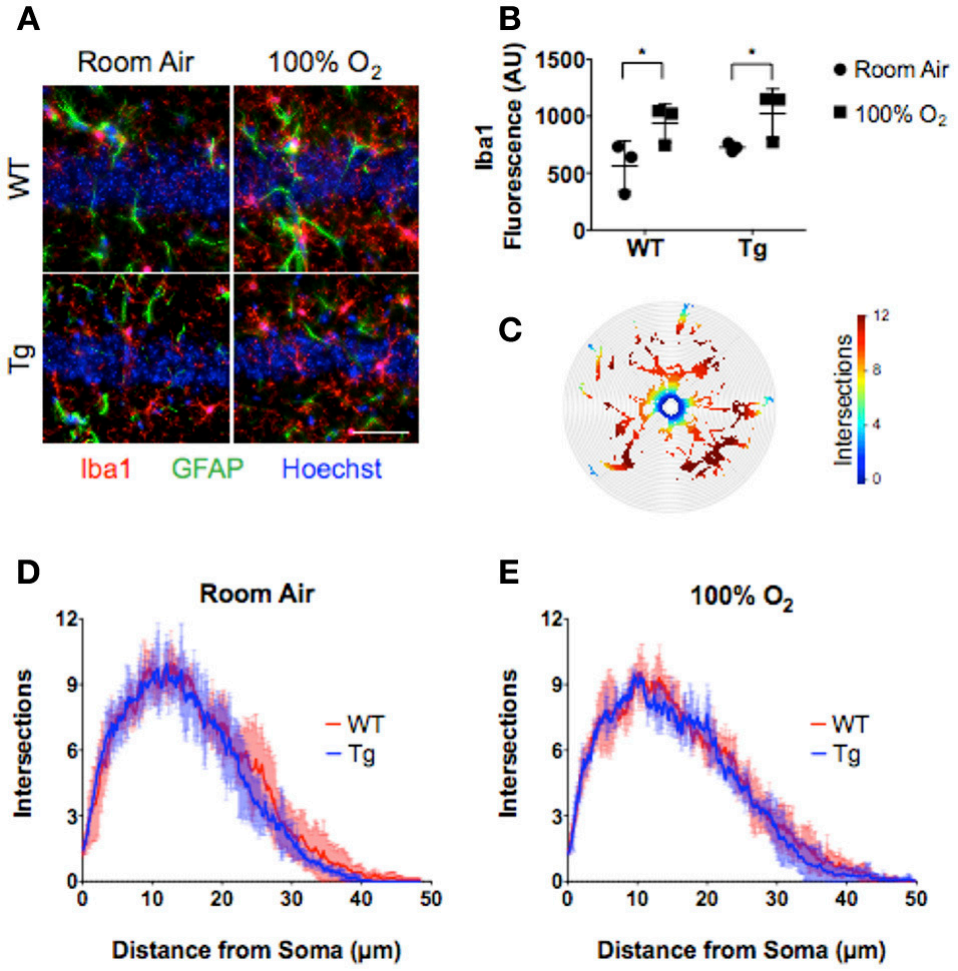
Over-expression of EC-SOD in the lung does not block hyperoxia-induced microglial priming in CA1 of the adult hippocampus. **(A)** CA1 field of the hippocampus showing neuronal nuclei, Iba1^+^ microglia, and GFAP^+^ astrocytes. Scale bar = 200 μm. **(B)** Neonatal hyperoxia stimulates Iba1 expression in both WT and EC-SOD mice; ^*^P < 0.05. **(C)** Sholl analysis heat map of microglial processes illustrating the number of intersections with concentric circles from the soma to most distal branch. **(D,E)** Sholl analyses demonstrating no differences in microglial arborization in either adult WT or *Sftpc*^*EC*−*SOD*^ transgenic mice exposed to room air or neonatal hyperoxia. (*n* = 4–5 mice per group except panel B where *n* = 3 mice per group). Data from individual mice are represented as circle (room air) or squares (hyperoxia) in **(B)**.

Neonatal hyperoxia induces inflammatory changes in the CNS marked by elevated cytokine production, reactive astrogliosis, and microglial activation (15). To assess the effects of lung EC-SOD expression on hyperoxia-induced neuroinflammation, we stained hippocampal brain sections from each cohort of female mice for the expression of the astrocyte marker GFAP and macrophage/microglial marker ionized calcium-binding adapter molecule 1 (Iba1). Neither hyperoxia (*p* = 0.318) nor genotype (*p* = 0.438) had an effect on GFAP expression (Figure 4) associated with injury-induced astrogliosis within the CA1 field of the hippocampus. Hyperoxia did induce the expression of Iba1 (WT, 563 ± 219 vs. 939 ± 166; Tg 727 ± 36 vs. 1023 ± 216; *p* = 0.018) within CA1 microglia (Figures 4A,B) without affecting the extent of microglial arborization measured by Sholl morphometric analyses (Figures 4C–E). Notably, EC-SOD expression had no effect on either the induced expression of Iba1 or the basal morphology of microglia within the CA1 field.

**Figure 5:**
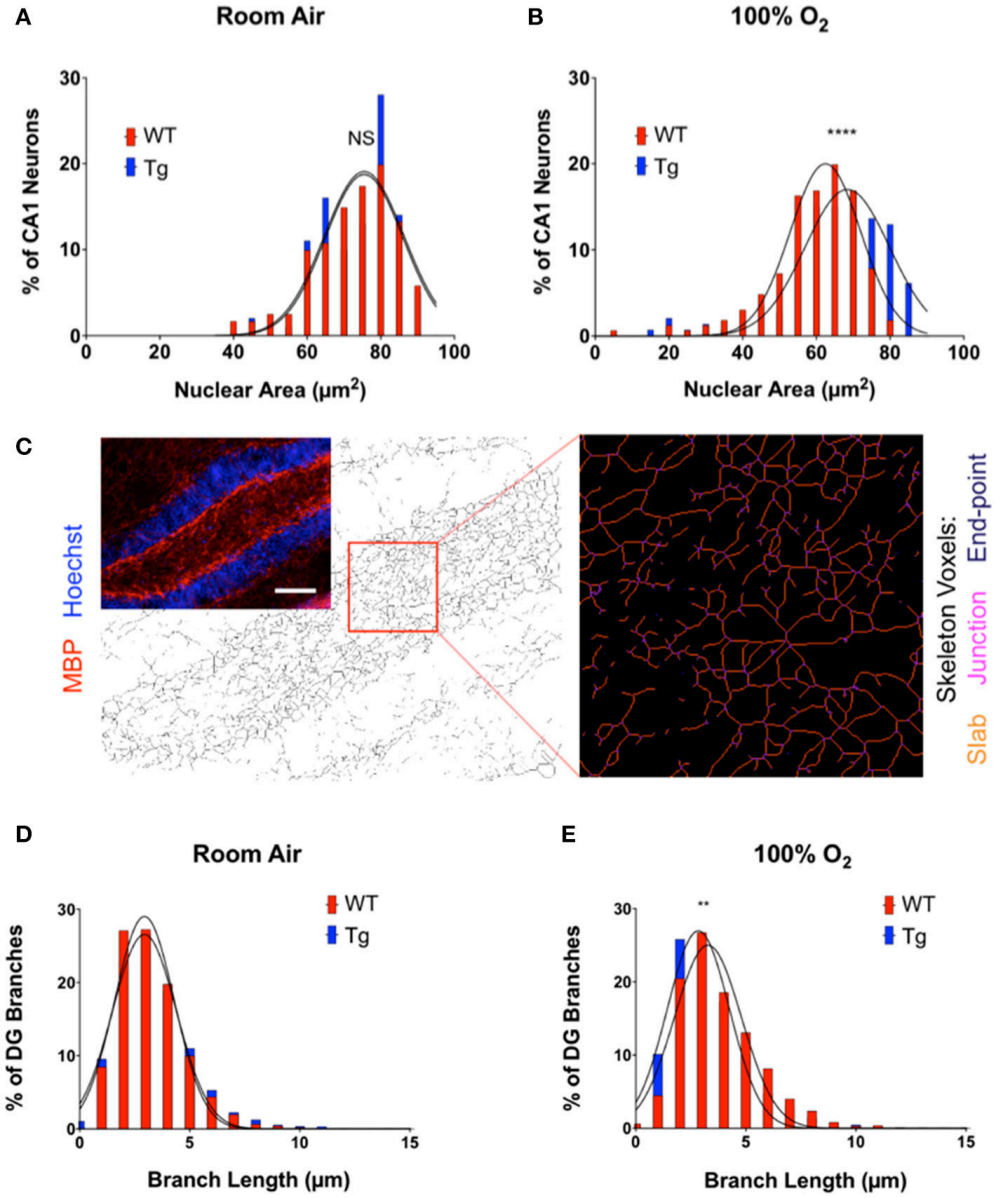
Over-expression of EC-SOD in the lung protects against hyperoxia-induced changes in neuronal morphology in CA1 and DG of the hippocampus. **(A)** Over-expression of EC-SOD has no effect on CA1 nuclear size in the CA1 field. Distribution and Gaussian curve fit of neuronal nuclear size in the CA1 field performed in room air-treated mice (WT, 72.80 μm^2^ ± 11.04 vs. EC-SOD, 73.83 μm^2^ ± 9.97; *p* = 0.74 for comparison of Gaussian curves). **(B)** Over-expression of EC-SOD in the lung blocks hyperoxia-induced nuclear shrinkage in exposed CA1 neurons (WT, 59.38 μm^2^ ± 12.09 vs. EC-SOD, 65.23 μm^2^ ± 14.06; ^****^p < 0.0001 for comparison of Gaussian curves). **(C)** Transgene expression of EC-SOD in the lung inhibits hyperoxia-induced fiber sprouting in the hilus of the dentate gyrus (DG). Skeleton analysis of MBP^+^ neurites in the DG hilus. Scale bar = 400 μm. **(D)** Histogram and Gaussian curve fit of branch lengths in the DG with no differences in room air-treated mice (WT, 3.23 μm ± 1.48 vs. EC-SOD, 3.36 μm ± 1.74; *p* = 0.27 for comparison of Gaussian curves). **(E)** Neonatal hyperoxia induces neurite sprouting in adult WT when compared to adult *Sftpc*^*EC*−*SOD*^ mice (WT, 3.79 μm ± 1.84 vs. EC-SOD, 3.33 μm ± 1.75; ^**^p < 0.01 for comparison of Gaussian curves). (*n* = 4–5 mice per group).

In addition to effects on neuroinflammatory markers, acute insults to the central nervous system can also cause cytoarchitectural changes within sub-regions of the hippocampus. Hippocampal neurons within the CA1 and DG are particularly sensitive to acute brain injury. The observed pathological responses include non-pyknotic, nuclear shrinkage as well as neuronal fiber sprouting, particularly within the dentate hilus and area CA3. To evaluate whether morphologic changes in these regions could explain the deficits with novel object recognition, brain sections from female mice were stained for myelin basic protein (MBP), a major component of the myelin sheath surrounding neurite fibers, and counterstained with Hoechst dye to assess nuclear size. In mice exposed to room air, over-expression of EC-SOD did not affect either nuclear number or area within the CA1 region (Figure 5A). Instead, a small but significant reduction in nuclear size was observed in adult mice exposed to neonatal hyperoxia, blocked by over-expression of EC-SOD in the lung (Figure 5B). Over-expression of EC-SOD also inhibited hyperoxia-induced sprouting of neuronal fibers within the hilus of the DG, without affecting fiber density in mice exposed to room air (Figures 5C–E).

## Discussion

It is generally accepted that the preterm lung is sensitive to oxygen-induced ROS and inflammation because it is deficient in anti-oxidant defenses. While the preterm brain is also sensitive to oxidative stress, it is not clear how supplemental oxygen used to treat preterm infants in respiratory distress damages the brain. Because the preterm brain is hyperoxygenated on the first day of life (34) and this is associated with poor neurological outcomes between ages 2 and 3 (8), ROS produced locally may injure the brain. However, arterial oxygen levels are tightly monitored in the clinic at the expense of pulmonary levels, suggesting that ROS produced in the lung may be responsible for injuring the brain. To address this question, we used a well-characterized mouse model of neonatal hyperoxia exposure that phenocopies adult diseases attributed to preterm birth, namely altered lung development, host response to respiratory viral infection, and cardiovascular health. We provide evidence that lung-specific overexpression of the anti-oxidant EC-SOD preserves short-term memory in adult mice exposed to hyperoxia as neonates. Surprisingly, it did not blunt oxygen-induced neuroinflammation as defined by increased expression of Iba1 in hippocampal microglia. Since ROS produced in the lung injure the developing brain, anti-oxidant therapies intended to preserve the developmental programming of the preterm lung may concomitantly provide some protection to the developing brain.

Analogous to the cognitive impairments observed in children born preterm (35), we found that neonatal hyperoxia altered memory, as assessed by novel object recognition, in adult wild type mice exposed to hyperoxia as neonates. Our finding is consistent with another study showing how exposure of newborn mice to 85% oxygen between postnatal days 1–14 causes deficits in spatial and recognition memory associated with smaller hippocampal sizes in adults (12). We extended these findings by showing that a shorter duration of neonatal hyperoxia, namely one that parallels the saccular phase of lung development when most preterm infants are treated with supplemental oxygen, can also alter metrics of neurodevelopment, specifically memory. Furthermore, it does so through a pathway that is protected by lung-specific overexpression of EC-SOD in mice. While it is possible that an elevated arterial PaO_2_ promotes changes in neurodevelopment through a direct pathway involving hyperoxia-induced brain injury via oxidative stress, our findings support the contribution of an indirect pathway involving an interaction between the lung and the brain. Clinical evidence for such an interaction is seen in adults with acute respiratory distress syndrome (ARDS) and exposed to supplemental oxygen, who show persistent and progressive cognitive deterioration upon discharge (36). Mechanical ventilation used to treat ARDS patients promotes inflammation and the production of pro-inflammatory mediators, which are thought to propagate systemically through a yet to be determined mechanism and potentiate injury to peripheral organs, such as the brain, where the inflammatory signal can become amplified (37).

It is unlikely that ROS, especially superoxide produced in the lung can circulate and toxify the developing brain because they are simply too reactive. A more likely scenario is that lung-derived ROS stimulate a local inflammatory response that circulates to the brain. Consistent with this idea, we recently showed that neonatal hyperoxia (60% oxygen between PND1 and PND4), followed by exposure to concentrated ambient ultrafine particles or CAPS (between PND4-7 and PND10-13) produces greater deficits in adult learning than either insult individually (38). This interaction between hyperoxia and CAPS was not seen when neonatal mice were exposed to 100% oxygen, such as used in the current study, which caused significantly greater changes in the brain than the combined lower dose of oxygen and CAPS. Since air pollution drives inflammatory lung injury, these findings suggest a cumulative dose of pulmonary inflammation during critical early stages in brain development can adversely impact cognitive performance later in life. Interestingly, receptors for pro-inflammatory mediators are distributed throughout the brain, with the hippocampus having one of the highest densities (39). Experimental studies have shown in various animal models that acute lung injury and systemic inflammation provoke damage in the CNS, with the hippocampus being one of the most vulnerable regions (40, 41). It is known that the hippocampus is essential for learning, memory, and cognition. In fact, the correlation between hippocampal atrophy and deficits in learning have recently been demonstrated in adult mice exposed to hyperoxia as neonates (12). Regardless, anti-oxidants appear to be effective at alleviating oxygen-induced injury to the developing brain. Oxygen-dependent apoptosis in the hippocampus and cerebellum, and protein markers of neuroinflammation were attenuated in 1 week old transgenic mice that ubiquitously over-expressed EC-SOD under the actin promoter (24). This supports the idea that EC-SOD can block neuronal injury and regional inflammation but does not address whether it does so by detoxifying ROS in the lung or brain. Our research found that lung-specific EC-SOD preserves memory in adults without blocking microglia activation. It suggests ROS produced in the lung cause long-term changes in cognition independent of persistent microglia activation but does not address how it influences neurologic apoptosis or whether microglia play a role during exposure.

Microglia play an important role in maintaining brain homeostasis under conditions of ischemia-reperfusion injury, infection, and other insults (42). For example, they produce neurotrophic factors that aid in cellular repair and recruit immune cells into the brain to clear infection (43). Conversely, under conditions of chronic inflammation, activated microglia can be harmful and are thought to contribute to the pathogenesis of several neurodegenerative diseases, such as Parkinson's disease and Alzheimer's (44). The underlying factors regulating microglial polarization remain unclear, and changes in microglial morphology do not faithfully reflect changes in their activation or function. This complicates interpreting their activation state simply based upon Iba1 staining. One concept that has been considered is that of microglial priming, in which cells become sensitized by some insult or injury during development, and their later response to some challenge becomes exaggerated (45). While primed microglia are morphologically similar to activated microglia (ameboid), they do not chronically produce cytokines or other pro-inflammatory mediators that are capable of eliciting inflammation (43). However, they will produce these factors upon a subsequent insult, such as an infection, and overproduction of such factors may lead to adverse neurological complications (46). In the present study, neonatal hyperoxia increased expression of ionized calcium binding adaptor molecule 1 (Iba1), a protein thought to play a role in microglial activation and function, in both wild type and *Sftpc*^*EC*−*SOD*^ mice. Since short-term memory was preserved in *Sftpc*^*EC*−*SOD*^ mice exposed to hyperoxia as neonates, it is possible that the increased Iba1 expression observed in the ameboid microglia of these mice, as well as the WT mice, reflect primed microglia, as opposed to truly activated microglia. Alternatively, the increased Iba1 expression in the microglia of WT and EC-SOD transgenic mice is indicative of microglial priming, rather than activation. In this case, one could posit that the loss of one or more adaptive functions by microglia could explain the cognitive deficits observed. Conversely, hyperoxia could transiently induce a toxic gain-of-function in exposed microglia, which could wane over time with resolution of other inflammatory processes. Either way, the fact that we did not observe reactive astrogliosis and increases in GFAP expression argues that the cumulative effects on the CNS in our model are relatively mild. Further studies will be required to definitively test these competing hypotheses. Our findings also beg the question whether primed Iba1 non-ameboid microglia may sensitize the brain to subsequent insult or might contribute in some way to age-related neurodegeneration.

We also observed several morphological changes specific to hippocampal neurons that may underlie the observed neurocognitive deficits seen in hyperoxia-exposed subjects. Despite their selective vulnerability in acute neurological injuries, hyperoxia exposure did not result in frank CA1 neuronal loss. We did note an average reduction in the size of CA1 neuronal nuclei, consistent with the response to mild injury seen after both ischemia and excitotoxic injury (31, 47). Hyperoxic exposure also triggered fiber sprouting in the hilus of the DG, which is notable given the association between sprouting and the development of spontaneous recurrent seizures. How then do we resolve the gender specific differences observed on the tests of novel object recognition? In females, 17β-estradiol levels correlate directly with the synthesis of the neurotrophin BDNF within mossy fiber projections, while testosterone exerts tonic suppression of BDNF in males (48). As predicted, testosterone depletion in males has also been shown to increase mossy fiber sprouting in area CA3 of the hippocampus (49). This link between sex hormones, BDNF activity and hippocampal plasticity is also proposed to explain the higher incidence of anxiety related disorders and PTSD in women compared to men (48). Thus, one could deduce that the female brain may be inherently more sensitive to hyperoxia-induced pathological neuroplasticity than the male brain, resulting in the observed pathological and behavioral changes.

In summary, we have shown that EC-SOD targeted to the respiratory epithelium of mice protects against early postnatal changes in alveolar development during hyperoxia, and that this protection is associated with a preservation of short-term memory. The findings presented here support the use of neonatal hyperoxia as a model for continued investigation of potential pathways contributing to neurodevelopmental impairment. The notion that ROS produced in the lung are responsible for initiating, perpetuating, or promoting neurologic disease helps focus research on identifying the source of ROS that perturbs brain development, such as in infants born preterm. If related to human disease, anti-oxidant therapies designed to improve respiratory health may also be efficacious in concomitantly providing protection to the developing brain.

## Author Contributions

BB designed experiment, exposed mice to hyperoxia, assisted learning and locomotor studies, tissues processing, western blotting, interpreted data, reviewed and wrote manuscript. NM tissue processing and staining, reviewed manuscript. MY exposed mice to hyperoxia, tissue processing, reviewed manuscript. JA performed learning and locomotor studies, reviewed manuscript. LP tissue processing and staining, reviewed manuscript. DC-S designed experiments, interpreted data, reviewed and wrote manuscript. MH designed experiments, interpreted data, reviewed and wrote manuscript. MO designed experiments, interpreted data, reviewed and wrote manuscript.

### Conflict of Interest Statement

The authors declare that the research was conducted in the absence of any commercial or financial relationships that could be construed as a potential conflict of interest.
